# Sharp-Wave Ripple Frequency and Interictal Epileptic Discharges Increase in Tandem During Thermal Induction of Seizures in a Mouse Model of Genetic Epilepsy

**DOI:** 10.3389/fncel.2021.751762

**Published:** 2021-10-18

**Authors:** Christine S. Cheah, Megan A. Beckman, William A. Catterall, John C. Oakley

**Affiliations:** ^1^Department of Neurology, University of Washington, Seattle, WA, United States; ^2^Department of Pharmacology, University of Washington, Seattle, WA, United States

**Keywords:** Dravet syndrome, sharp-wave ripple, interictal epileptiform discharge, febrile seizure, channelopathies, mouse model

## Abstract

Dravet Syndrome (DS) is a genetic, infantile-onset epilepsy with refractory seizures and severe cognitive impairment. While network level pathophysiology is poorly understood, work in genetic mouse models of DS reveals selective reduction of inhibitory interneuron excitability, a likely mechanism of seizures and comorbidities. Consistent with the critical role of interneurons in timing and recruitment of network activity, hippocampal sharp wave ripples (SPW-R)—interneuron dependent compound brain rhythms essential for spatial learning and memory—are less frequent and ripple frequency is slower in DS mice, both likely to impair cognitive performance. Febrile seizures are characteristic of DS, reflecting a temperature-dependent shift in excitation–inhibition balance. DS interneurons are sensitive to depolarization block and may fall silent with increased excitation precipitating epileptic transformation of ripples. To determine the temperature dependence of SWP-R features and relationship of SPW-R to hippocampal interictal activity, we recorded hippocampal local field potentials in a DS mouse model and wildtype littermate controls while increasing core body temperature. In both genotypes, temperature elevation speeds ripple frequency, although DS ripples remain consistently slower. The rate of SPW-R also increases in both genotypes but subsequently falls in DS mice as interictal epileptic activity simultaneously increases preceding a thermally-evoked seizure. Epileptic events occur intermixed with SPW-R, some during SPW-R burst complexes, and transiently suppress SPW-R occurrence suggesting shared network elements. Together these data demonstrate a temperature dependence of SPW-R rate and ripple frequency and suggest a pathophysiologic mechanism by which elevated temperature transforms a normal brain rhythm into epileptic event.

## Introduction

Mutations in *SCN1A*, the gene encoding the type I voltage-gated sodium channel Na_v_1.1, are associated with a family of epilepsies in which febrile seizures are the minimum epileptic phenotype (Catterall et al., 2010; Scheffer and Nabbout, 2019). The most severe of the *SCN1A* related epilepsies is Dravet Syndrome (DS), an early onset, pharmacoresistant epilepsy associated with complete loss-of-function mutations leading to Na_v_1.1 haploinsufficiency and characterized by multiple seizure types, including lifelong sensitivity to thermally evoked seizures, persistent cognitive impairment, and other behavioral comorbidities (Dravet, 2011).

Mouse models of DS haploinsufficient for *Scn1a* are accurate genotypic and phenotypic mimics (Yu et al., 2006; Ogiwara et al., 2007). *In vitro* electrophysiologic studies in DS mice reveal reduced excitability and decreased firing of inhibitory interneurons, while excitatory pyramidal cells are largely unaffected (Yu et al., 2006; Kalume et al., 2007; Tai et al., 2014). These deficits in inhibitory neurons impair network oscillations both *in vivo* and *in vitro* (Schlingloff et al., 2014; Cheah et al., 2019).

Hippocampal sharp wave ripples (SPW-R) are compound brain oscillations comprised of a high-frequency ripple oscillation superimposed on a slower, large amplitude sharp wave (SPW) and are essential for spatial learning (Buzsáki, 2015). Ripple frequencies are positively correlated with SPW-mediated excitation (Cheah et al., 2019) and both the rate of SPW-R occurrence and the frequency of the ripple oscillation within each SPW-R complex are increased following periods of learning (Ponomarenko et al., 2008). During a SPW-R, network excitability is transiently increased with strong, reliable inhibitory transmission synchronizing excitatory cell firing at the ripple frequency (Schlingloff et al., 2014; Ognjanovski et al., 2017). However, as predicted from their impaired inhibition, SPW-R in DS mice occur less frequently and with slowed ripple frequency (Cheah et al., 2019). Na_v_1.1 is the primary sodium channel in inhibitory neurons and haploinsufficiency in DS makes inhibitory neurons susceptible to entering depolarization block following a build-up of inactivated sodium channels during trains of impulses (Catterall, 2000; Yu et al., 2006; Tai et al., 2014) and complete failure of inhibition could lead to an avalanche of recurrent excitation among highly interconnected pyramidal cells, transforming SPW-R into epileptic discharges (Ziburkus et al., 2006).

The impact of elevated temperature on excitability and network behavior is difficult to predict (Qu et al., 2007; Hill et al., 2011; Kim and Connors, 2012; Van Hook, 2020) but, induction of seizures with fever in DS supports a temperature-dependent overall increase in hippocampal network excitability (Yu et al., 2006; Oakley et al., 2009). Changes in SPW-R frequency, SPW amplitude, rates of SPW-R occurrence, and interictal activity are further measures of network excitability. In this study, we measured these SPW-R features to test the hypothesis that temperature increases hippocampal network excitability and that this increase in excitability is less well controlled in epileptic networks.

## Materials and Methods

### Mouse Strains

*Scn1a* mutant mice were generated by targeted deletion of the last exon of *Scn1a* encoding domain IV, including from the S3-S6 segments and the entire C-terminal tail of Na_V_1.1 channels (Yu et al., 2006). Male and female *Scn1a*^+/–^ mutant (DS) mice and wild type (WT) littermates were generated by crossing heterozygous *Scn1a*^+/–^ males on C57BL/6J background with WT C57BL/6J females. Genotype was confirmed using a four-oligonucleotide multiplex PCR of genomic DNA (Yu et al., 2006). All experiments were performed according to guidelines of the National Institutes of Health National Research Council, 2011, and were approved by the University of Washington Institutional Animal Care and Use Committee.

### Surgery

Naïve, adult mice (WT *n* = 5; DS *n* = 7; ≥ postnatal day 30) were housed in standard housing cages in the University of Washington Animal Facility under specific pathogen-free (SPF) conditions with constant temperature and 12-h light/12-h dark cycle. Perfluoroalkoxy (PFA) insulated stainless steel wire electrodes (A-M Systems, Sequim, WA, 791000; diameter: 76.2 μm bare; 139.7 μm coated) were implanted aseptically using stereotactic guidance (David Kopf Instruments, Tujunga, CA) through small burr holes under ketamine/xylazine (130/8.8 mg/kg) anesthesia and targeted to bilateral dorsal hippocampus using standard stereotactic coordinates (Bregma: -1.75 mm, Dorsal-Ventral: -1.1 mm, Lateral: -1.25) then fixed in place with dental cement (Cheah et al., 2019). To simultaneously record sharp waves and ripple components, electrodes were targeted to proximal stratum radiatum, and locations were confirmed by characteristic electrophysiologic patterns and histology (Cresyl violet Nissl stain, 50 m slices, 4% PFA transcardial perfusion; Cheah et al., 2019). Screw electrodes over the cerebellum served as ground and reference. Electromyogenic (EMG) activity was recorded from stainless steel fine wire electrodes placed in bilateral neck extensor muscles. Mice recovered for >48 h prior to recordings and animals were singly housed in standard home cages until completion of the local field potential recordings.

### Local Field Potential Recordings and Thermal Induction

Local field potential (LFP) recordings were made in a plexiglass recording chamber during the light phase of the day and core body temperature was monitored *via* battery powered rectal thermocouple to reduce electrical noise (Physitemp Instruments LLC, Clifton, NJ 07013). A digital recording system (Tucker-Davis Technologies, Alachua, FL 32615) recorded hippocampal LFP and neck extensor muscle EMG signals oversampled at 6 KHz and subsequently digitally filtered offline at 1,024 Hz and downsampled to 2048 Hz for maximal flexibility in filter design and signal processing. Body temperature was recorded at 10 Hz. A modified thermal induction protocol (Oakley et al., 2009) using indirect exposure to a heat lamp slowly increased each animal’s core body temperature from baseline to seizure or a maximum core body temperature of 40°C. The animal was then allowed to passively cool before being returned to its home cage.

### Offline Signal Analysis and SPW-R Detection

Individual SPW-R identification was performed offline using an automated approach and algorithms developed in-house (Igor, WaveMetrics; Cheah et al., 2019). Signals were bandpass filtered (finite impulse response (FIR), zero-lag, linear phase, and Hanning window; EMG 200–700 Hz, Ripple 100–260 Hz, Sharp wave 10–20 Hz), the instantaneous amplitude was computed by Hilbert transform and squared to estimate instantaneous power. Z-score normalization of instantaneous power was used to control for differences in amplitude and signal-to-noise across recording sites and mice where:


$$z-score\;=\;\frac{\left(f(t)-\bar{x}\right)}{\sigma }$$

*f*(*t*) = recorded signal, $\bar{x}$ = arithmetic mean, and σ = standard deviation.

Mean and standard deviation of a representative period of NREM sleep were used to minimize the impact of movement and myogenic artifact and emphasize variance of SPW-R which constitute a greater proportion of the record in sleep. NREM sleep was identified by low EMG power, LFP high voltage irregular activity, and absence of theta gamma activity.

The behavioral state was identified by instantaneous 200–700 Hz normalized power in EMG recording. Active exploration was identified by EMG power >1–2 SD, adjusted as needed to ensure periods of characteristic exploratory theta gamma activity were appropriately classified. REM sleep was not observed during brief baseline recordings or at elevated body temperature during thermal induction. Our prior studies (Cheah et al., 2019) did not show significant differences in SPW-R features between waking immobility and NREM sleep and in the present work, awake immobility was predominant and was combined with periods of NREM sleep for analysis.

Candidate SPW-R epochs were identified by increases in normalized, instantaneous ripple band power >3 SD. Detections during periods classified as active exploration were excluded. Within each epoch, peaks and troughs were identified in the ripple bandpass filtered recording excluding peak-trough pairs detected >10 or <4 ms (corresponding to 100–260 Hz) from the last identified pair, typically false detections. The start and end times of each ripple were determined by the first and last troughs within each candidate epoch. For each detected SPW-R, the five middle peak-trough cycles (three for ripples containing fewer than six cycles) were used to determine average ripple frequency from inter-trough interval duration, average and maximal ripple power in the ripple band normalized power transformed signal, and average and maximal SPW power from the SPW band normalized power transformed signal.

### Interictal Spike Detection

Interictal spikes were identified by two characteristic features in hippocampal LFP recordings: high amplitude, and fast kinetics. As with SPW-R detection, differences in recording amplitude and signal-to-noise were addressed through z-score normalization using mean and SD of an identified period of NREM sleep. Candidate interictal spikes were identified by simultaneous high amplitude (>1 SD) and steep first derivative (>5 SD). Detected spikes were reviewed and artifacts, identified by unusual morphology and movement artifact in multiple channels, were rejected. Myoclonic seizures were separable from interictal spikes by a transient increase in myogenic activity immediately following one or often multiple preceding spikes. EMG normalized power was determined 100 ms prior to and 100 ms following spike peak with a post to pre-spike power ratio >2 consistent with myoclonus.

### Statistics

Linear correlation analysis was used to determine the statistical significance of the covariance of continuous measures (Pearson’s R, α = 0.05). Differences in group means were determined by *t*-test.

## Results

### Frequency-Based Power Analysis in the SPW-R Band

**Figure 1:**
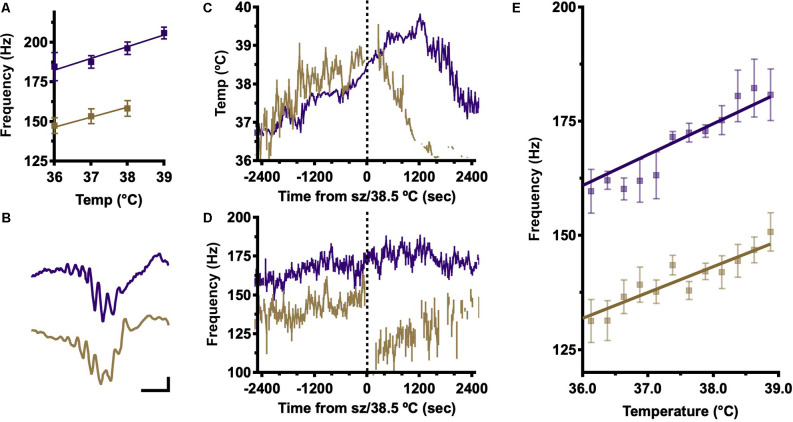
Frequency analysis shows an increase in SPW-R frequency with temperature elevation. **(A)** Frequency at peak ripple band (100–260 Hz) power, determined in average power spectra of hippocampal LFP signals in 1°C temperature bins, shows increasing ripple frequency with temperature in both genotypes but slower frequency in DS across all temperatures. Frequencies were calculated for each mouse and then the grand-average frequency was determined by genotype to generate means ± SEM. **(B)** Examples of individually detected SPW-R in WT (purple) and DS (gold) mice. Calibrator 200 microvolts, 20 ms. **(C)** Average body temperature (±SEM) aligned on seizure (DS, gold) or 38.5°C (WT). The temperature profile does not differ between genotypes. **(D)** Average ripple frequency (±SEM) aligned on time of GTC in DS mice (gold) or on time of crossing average DS seizure temperature 38.5°C in WT (purple). **(E)** Ripple frequency was estimated for each detected SPW-R and averaged by temperature (0.25°C bins) for each mouse then a grand average was determined by genotype. A positive correlation between temperature and frequency of individually detected SPW-R is present in 6/6 DS and 5/5 WT mice (linear correlation Pearson’s R, α = 0.05), but DS SPW-R is slower at all temperatures (genotype grand average ± SEM). SPW-R, hippocampal sharp wave ripples; DS, Dravet Syndrome; LFP, Local field potential; GTC, generalized tonic-clonic.

To estimate the dependence of internal ripple frequency on body temperature, local field potential recordings were made using chronic, stereotactically implanted fine wire electrodes in dorsal CA1. After a period of baseline recording, body temperature was slowly increased until a seizure occurred (all DS mice) or, in the absence of seizures, a body temperature of 40°C was achieved (all WT mice). Offline, periods of inactivity reflecting awake immobility and NREM sleep were concatenated by temperature in 1°C increments and average frequency spectrograms generated for each. Ripple frequency was estimated at each temperature by Gaussian fit to spectral power in the ripple band (100–260 Hz; Figure 1A). This analysis shows increasing ripple frequency with temperature up to 39°C, when a seizure has occurred in most DS mice. These findings are consistent with our prior study of SPW-R in DS mice in which DS SPW-R are slower at baseline (Cheah et al., 2019; Figure 1A; 36°C; DS, 135 Hz vs. WT, 175 Hz). In both genotypes, ripple frequency increases with temperature; however, DS ripples remain slower across the entire temperature range.

### Individual SPW-R Features During Temperature Induction

To confirm the temperature dependence of internal ripple frequency and determine the impact of temperature on other SPW-R features, individual SPW-R events were identified offline (Cheah et al., 2019; Methods). Ripples are identifiable by a brief, substantial increase in 100–260 Hz power above baseline reflecting a transient increase in highly synchronous neuronal activity, and candidate ripples were characterized through the detection of peaks and troughs. Average SPW-R were aligned on the ripple trough nearest the SPW peak. Individually detected SPW-R have similar morphology in DS and WT across temperatures (Figure 1B), confirming appropriate SPW-R detection. Average body temperature was determined for each detected SPW-R and plotted as a function of time from the onset of generalized tonic-clonic (GTC) seizures in DS mice (39.0 ± 0.1°C; range 38.4–39.4°C; Figure 1C gold). WT mice never have seizures; thus, their temperature profiles were aligned to the time that core body temperature reached 38.5°C, the published temperature of seizure onset in DS, and the low end of the seizure temperature range in DS mice from this study (Figure 1C, purple; Oakley et al., 2009). WT and DS mice have overlapping temperature trajectories during thermal induction; therefore, differences in peak ripple band frequency cannot be explained by differences in the thermal induction profile.

Average instantaneous ripple frequency was estimated for each SPW-R from inter-trough intervals and then aligned by time from seizure (DS) or crossing 38.5°C (WT; Figure 1D). Immediately following generalized tonic-clonic (GTC) seizure onset there is a dramatic reduction in internal SPW-R frequency in DS mice, marking a significant difference in pre-ictal and post-ictal frequency modulation, which is not observed in WT above 38.5°C (Figure 1D). Ripple frequencies from individually detected SPW-R complexes in the pre-ictal range of the temperature induction were binned in 0.25°C bins (Figure 1E). Consistent with the findings from frequency-based analysis (Figure 1A), the average internal ripple frequency of individually detected SPW-R complexes increases with body temperature, and at each temperature, the frequency is always slower in DS (Figure 1E). These results indicate that temperature is a sufficient stimulus for increasing ripple frequency and that epileptic activity disrupts SPW-R generating mechanisms.

### SPW Features During Temperature Elevation

SPW-R recorded in CA1 are a compound oscillatory signature composed of a local fast ripple oscillation in CA1 superimposed on a higher amplitude sharp wave reflecting synchronous excitation from CA3 pyramidal cells targeting CA1 stratum radiatum. As such, the parallel increases in internal SPW-R frequency and temperature could be a result of local, intrinsic temperature-related changes in CA1 network properties or a temperature-related increase in the excitatory drive.

**Figure 2:**
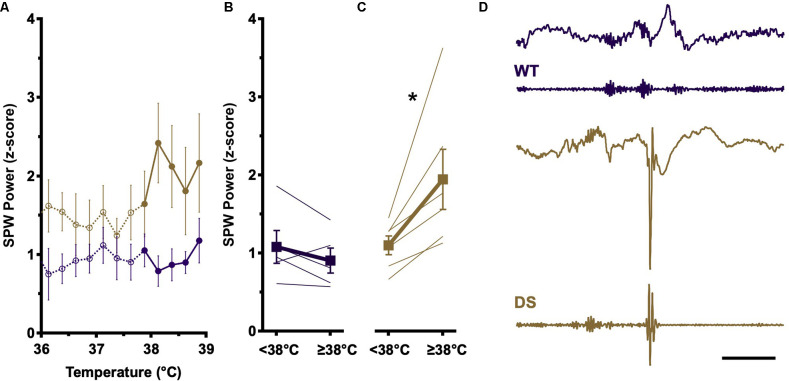
SPW amplitude increases at higher body temperatures in DS mice. **(A)** Increases in SPW (presented as z-score ± SEM) are comparable at baseline temperatures (<38°C) in WT (open purple circles) and DS (open gold circles) SPW-R. At elevated temperatures (≥38°C), SPW amplitude increases only in DS (closed gold circles), whereas WT distributions remain similar to baseline (closed purple circles). **(B)** SPW amplitudes from individual mice and genotype grand average (±SEM) above and below 38°C in WT (purple) are not statistically different (paired *t*-test, *p* > 0.05). **(C)** SPW amplitudes from individual mice and genotype grand average (±SEM) above and below 38°C in DS (gold) are shifted to higher SPW amplitude at elevated body temperature (paired *t*-test, *p < 0.05) WT *n* = 5; DS *n* = 6. **(D)** Example of spike occurrence during SPW-R burst complexes. LFP (top) and bandpass filtered (FIR 100–300 Hz, bottom) traces of SPW-R activity in WT (purple) and DS (gold) mice. WT example shows burst complex of three SPW-R. DS example shows a SPW-R followed by a spike and subsequent attenuation of faster frequencies in LFP signal, characteristic of epileptic discharges. Calibration, 200 ms. WT, Wild type; FIR, finite impulse response.

The amplitude of SPW recorded in CA1 reflects the degree of activation of CA3 pyramidal cells (Ylinen et al., 1995). To determine if temperature elevation impacts CA3 network properties, we analyzed the SPW component of detected SPW-R during the pre-ictal phase of temperature induction. Consistent with prior reports (Cheah et al., 2019), at baseline temperatures (<38°C), DS and WT SPW distributions are overlapping (Figure 2A, open circles). WT SPW amplitudes remain constant across the entire temperature range (Figure 2A, purple, open vs. filled) and no difference is seen between SPW amplitudes above and below 38°C (Figure 2B). In contrast, in DS recordings average SPW amplitudes increase substantially above 38°C (Figure 2A, gold, open vs. filled), and this increase in SPW amplitude is present in all DS recordings above 38°C (Figure 2C).

The overlapping SPW distributions at low and high temperatures in WT recordings suggest that temperature modulates intrinsic network properties in CA1, effectively adjusting the gain of ripple coupling to SPW such that a similar level of SPW excitation results in a higher SPW-R internal frequency at elevated temperatures (Figure 1E). However, in DS, higher amplitude SPW are seen above 38°C, reflecting an increase in the excitatory drive from CA3 which could overwhelm the impaired inhibition in CA1 observed in DS mice. SPW-R events typically occur in burst complexes in which three or four individual SPW-R events occur within 100–200 ms (Figure 2D, top). During some DS mouse burst complexes, following one or more SPW-R a spike occurs at the expected time of the next SPW-R, suggesting either disruption of SPW-R by the spike or epileptic transformation of SPW-R (Figure 2D, bottom). The loss of Na_V_1.1 in DS makes inhibitory neurons susceptible to failure. At elevated temperatures increased drive from CA3, in the form of higher-amplitude SPW, could overwhelm these inhibitory networks, leading to depolarization block and large excitatory discharge or spikes.

### Rate of Occurrence of SPW-R and Interictal Activity With Increasing Temperature in DS

**Figure 3:**
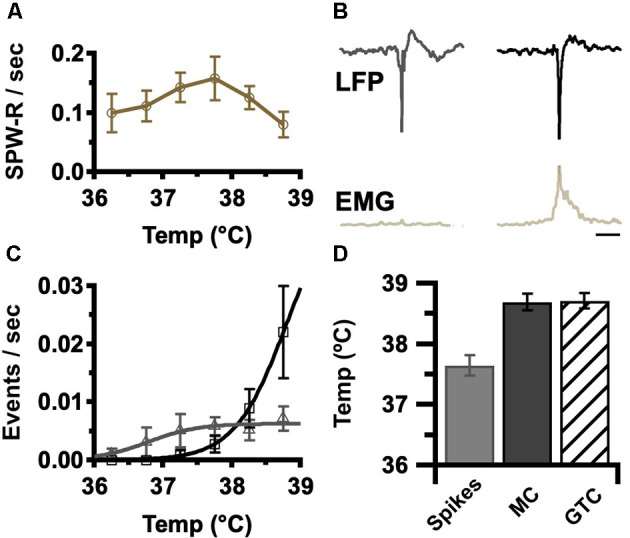
Occurrence of epileptic events increases with core body temperature. **(A)** Average rate of SPW-R generation was determined for the indicated temperatures in 0.5°C bins (±SEM). Grand average by genotype, DS, gold *n* = 6. The rate was determined by the number of detected events divided by time at temperature in each bin. Sigmoid fits show trends. **(B)** Average LFP and instantaneous EMG RMS power aligned to the highest amplitude spike trough for interictal spikes (left) and myoclonic seizures (MC, right). Interictal spikes were differentiated from MC by brief EMG activity. Calibration, 200 ms. **(C)** Average spike (gray), and MC (black) rate was determined by the temperature in 0.5°C bins (±SEM). As the MC rate increases (panel **C**, black), SPW-R rate declines (panel **A**). Grand average by genotype, DS *n* = 6. The rate was determined by the number of detected events divided by time at each temperature. Sigmoid fits show trends. **(D)** Interictal spikes occur at a lower temperature (37.8 ± 0.2°C) than MC (38.8 ± 0.2°C) and generalized tonic-clonic seizures (GTC; 38.9 ± 0.2°C). Grand average, *n* = 6 DS mice, spikes vs. MC *p* = 0.002, spikes vs. GTC *p* = 0.002, MC vs. GTC *p* = 0.9. EMG, electromyogenic.

Elevated temperature in DS elicits a progression of ictal activities, beginning with electrographic interictal spikes devoid of behavioral correlate, progressing to myoclonic seizures (MC), brief behavioral and electrographic events, until culminating in a large behavioral and electrographic generalized tonic-clonic seizure (GTC; Oakley et al., 2009; Liautard et al., 2013). The rate of occurrence of SPW-R in DS was determined for each temperature in the pre-ictal phase of temperature induction. These rates increase with temperature up to 38°C and then decrease prior to seizure onset (Figure 3A). The interictal activity detected in these recordings included both interictal spikes and myoclonic spikes. These activities were both typified by a similar high-amplitude spike component but could be distinguished by the occurrence of EMG activity above baseline associated with myoclonic spikes (Figure 3B). As expected, the rates of both of these forms of interictal activity increase with temperature (Figure 3C).

Surprisingly the peak rate of SPW-R (Figure 3A: 37.5°C peak for SPW-R) precedes the onset of seizures at 38.9 ± 0.2°C (Figure 3D) and is coincident with the increase in myoclonic spikes (compare Figures 3A–C). These results indicate that the rate of occurrence of SPW-R increases with temperature below the febrile range (Figure 3A: 37.5°C for peak SPW-R) but as temperatures approach fever (≥38°C), epileptic activity increases (Figure 3C) while the rate of SPW-R simultaneously declines (Figure 3A).

### Impact of Epileptic Activity on SPW-R Occurrence

**Figure 4:**
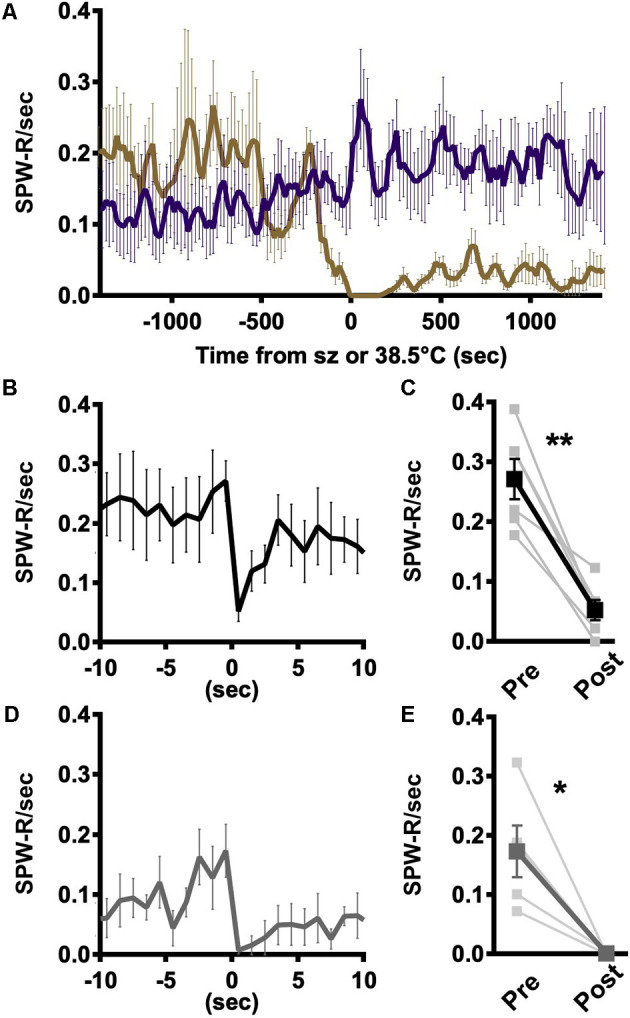
Rate of SPW-R is attenuated following spikes and seizures. **(A)** Rate of occurrence aligned to the time of seizure onset (DS, gold) or 38.5°C crossing (WT, purple) is similar in both genotypes and increases with temperature. Following the seizure, SPW-R occurrence is significantly reduced in DS while continuing to increase at comparable temperatures in WT. **(B)** Average instantaneous rate SPW-R aligned to spike trough shows SPW-R rate is briefly suppressed following spikes. **(C)** The rate of SPW-R in the 1 s following a spike is significantly reduced compared to 1 s prior to thes spike (paired *t*-test, **p < 0.005). **(D)** Following myoclonic seizures (MC), the instantaneous rate of SPW-R is suppressed. **(E)** The rate of SPW-R in the 1 s following MC is reduced as compared to the 1 s prior (paired *t*-test, *p < 0.05). **(B)** Spikes (black) and **(D)** MC (gray). Grand average *n* = 6 mice, 250 ms time bins.

The coincident decline in SPW-R occurrence and emergence of myoclonic events suggests that there is an interplay between epileptic activity and SPW-R (Figures 3A–C). However, the rates of occurrence are not on the same scale, making a one-to-one conversion unlikely. Local network excitability is known to be transiently suppressed following large epileptic discharges and therefore should be refractory to the generation of SPW-R (Bruno and Richardson, 2020). Consistent with that expectation, the rate of SPW-R occurrence declines precipitously following generalized tonic-clonic seizures in a way not seen with elevated temperatures in WT (Figure 4A). Individual hippocampal interictal spikes briefly suppress action potential firing in the hippocampus (Zhou et al., 2007). To determine if less severe epileptic events impacted SPW-R occurrence, the average instantaneous rate of SPW-R occurrence was aligned with either the trough of interictal spikes or myoclonus. Following interictal spikes, the rate of SPW-R occurrence is briefly reduced (Figure 4B), and in all recordings, a significant suppression is seen in the 1 s following an interictal spike (Figure 4C). A similar brief suppression is also observed following myoclonus (Figures 4D,E). In both cases, suppression is much less severe and of shorter duration than that observed following GTC (Figure 4A). Together these results demonstrate that SPW-R rates are suppressed proportional to the size of the epileptic event.

## Discussion

### Excitability of Hippocampal Network Activity Increases With Temperature

Elevated temperature is a stimulus for seizures and interictal activity in DS, but less is known regarding the impact of temperature on network oscillations, including SPW-R. Temperature modulates cellular and network function in many ways and the net impact on network function is difficult to predict *a priori*. Seizures induced by elevated temperature are characteristic of *SCN1A*—related epilepsies in general and DS in particular suggesting an overall net increase in excitatory to inhibitory balance. Temperature elevation *in vitro* and *in vivo* increases neuronal excitability through direct changes in biophysical properties of ion channels as well as increased firing rate (Kim and Connors, 2012). Increased pyramidal cell activity is predicted to promote SPW-R generation through the increased probability of excitation in CA3 sufficient to trigger an SPW (Buzsáki, 2015). Here we report SPW-R rate of occurrence increases with temperature in DS and WT mice consistent with a net increase in network excitability (Figure 3). When an SPW invades CA1 it excites pyramidal cells and GABAergic interneurons, including fast-spiking parvalbumin-expressing interneurons (Schlingloff et al., 2014; Stark et al., 2014; Buzsáki, 2015). Within CA1, synchronous inhibitory interneuronal activity paces high-frequency network oscillation in the form of ripples, with the internal frequency of the oscillation within a ripple proportional to the magnitude of SPW-mediated excitation (Schlingloff et al., 2014; Stark et al., 2014). SPW-R increase their internal ripple frequency and rate of occurrence during periods of greater excitatory drive, most notably following a novel experience, making it likely that elevated temperature would lead to an increase in ripple frequency (Ponomarenko et al., 2008).

The increase in internal ripple frequency seen here is consistent with these findings. Of note, there is no increase in SPW amplitude at baseline temperatures (<38°C) in either DS or WT animals (Figure 2), suggesting that the SPW-R generating networks in DS are functioning similarly to their WT counterparts and that temperature elevation changes the “gain” of the local CA1 response to the same amplitude SPW coming from CA3. A change in the input-output relationship in DS would be expected to appear as a change in the slope of the relationship between temperature and internal frequency, but the slowed frequency of SPW-R in DS is maintained across all temperatures (Figure 1).

### Large Amplitude SPW Appear Coincident With Increases in Interictal and Ictal Events

The timing of the ripple oscillation component of SPW-R is dependent on the reliable firing of “fast-spiking” parvalbumin (PV) basket cells (Schlingloff et al., 2014; Stark et al., 2014). Following learning, these cells increase firing rates to coordinate network activity (Ognjanovski et al., 2017). However, PV cells are also prone to failure during periods of repeat stimulation (Ziburkus et al., 2006; Tai et al., 2014; Magloire et al., 2019), and during periods of temperature elevation PV cells have been shown to enter depolarization block and cease firing (Kim and Connors, 2012). *In vitro* records of SPW-R in which hippocampal slices are presented with a strong excitatory stimulus show an initial increase in inhibitory neuron firing and parallel an increase in internal ripple frequency; however, inhibitory neurons eventually fail, leading to increased excitatory pyramidal cell firing and the emergence of high-frequency, ictal-like events (Ziburkus et al., 2006). Similarly, inhibitory cell firing increases preceding an ictal event, with cessation of firing or loss of coordinated firing immediately prior to ictal onset (Miri et al., 2018; Tran et al., 2020). Na_V_1.1 loss-of-function mutations responsible for Dravet Syndrome impact inhibitory neurons more severely than their excitatory counterparts, and these cells fail during periods of sustained activity (Yu et al., 2006; Tai et al., 2014). The increase in SPW amplitude could drive inhibitory neurons in the hippocampus to depolarization block and allow the transformation of SPW-R into interictal activity.

Hippocampal CA3 and CA1 neurons are differentially impacted by elevated temperature, with CA3 being more susceptible to heat-related biophysical changes and increased excitatory drive (Kim and Connors, 2012). Large amplitude SPW are detected only in DS mice above 38°C (Figure 2). Increased SPW amplitude in CA3 translates to increased excitatory drive to local CA1 networks, which would increase the firing rate of local networks and result in faster SPW-R internal frequency. However, increased excitation in CA1 could also drive PV-expressing and other classes of interneurons into depolarization block, leading to interictal and/or ictal activity (Ziburkus et al., 2006; Kim and Connors, 2012). Coincident with rising temperatures and increased SPW amplitude, epileptic spikes increase in rate but plateau prior to the onset of generalized tonic-clonic seizures. At the time of this plateau, the rate of myoclonic seizures increases while the rate of an SPW-R occurrence decreases (Figures 3A–C), and a SPW-R burst complex can be interrupted by an interictal spike (Figure 4C). These data are consistent with stochastic, high amplitude SPW overwhelming local CA1 inhibitory neurons and occasionally leading to a loss of inhibition and a high amplitude spike. The rate of SPW-R occurrence also increases with temperature (Figure 3). A temperature-dependent increase in CA3 excitability directly or increased excitability of CA2, an important initiator of waking SPW-R (Oliva et al., 2016), are possible factors. If the epileptic activity has a small probability of arising from each SPW-R, the increased rate will lead to more epileptic activity independent of any change in the probability of epileptic transformation.

### Predicted Impact of Temperature on Spatial Memory

SPW amplitude, ripple frequency, and SPW-R rate increase with learning (Ponomarenko et al., 2008) while the block of SPW-R impedes learning and memory (Girardeau et al., 2009). Recently we demonstrated reduced SPW-R rate and slowed ripple frequency in DS mice, likely contributing to impaired memory performance (Cheah et al., 2019). In the present work, SPW-R rate and ripple frequency in DS mice increase with temperature which in isolation would be predicted to improve memory. However, DS SPW-R fails to recover to WT levels prior to seizure. The reduced excitability of “fast-spiking” interneurons and their sensitivity to depolarization block may limit increases in SPW-R features and instead facilitate the transformation of normal SPW-R into interictal discharges.

Following spikes, and to a greater extent following seizures, cognitive performance is impaired (Power et al., 2018). Mechanisms leading to transient cognitive impairment following brief interictal spikes are unclear (Kleen et al., 2010; Faught et al., 2018) but could result from transient post-spike suppression of neuronal activity (Bruno and Richardson, 2020) previously demonstrated in the hippocampus (Zhou et al., 2007). Repetitive spikes with epileptic myoclonus and single interictal spikes generate a brief suppression of SPW-R, and substantially longer suppressed periods follow generalized tonic-clonic seizures (Figure 4), similar to previously reported reduction in single-cell firing activity following interictal discharges (Zhou et al., 2007). SPW-R occurrence varies with the excitability of CA3 pyramidal cells and it is possible that reduced SPW-R rate results from post-spike neuronal hypoexcitability and reduced probability of concurrent activation of CA3 pyramidal cells sufficient for initial SPW. Brief post-spike neuronal suppression and reduced SPW-R occurrence impacts cognitive function (Girardeau et al., 2009), providing a pathophysiologic mechanism for transient cognitive impairment observed in combination with interictal discharges (Faught et al., 2018).

## Summary

Our findings demonstrate that temperature elevation increases the rate of occurrence and internal ripple frequency of SPW-R in both wild-type and DS networks, with DS SPW-R consistently slower. In DS, the increase in the rate of occurrence and internal frequency of SPW-R are limited by the onset of epileptic activity, potentially reflecting the transformation of some SPW-R into interictal events. In this situation, interictal events can suppress SPW-R activity and directly contribute to the cognitive comorbidities in DS.

## Data Availability Statement

The raw data supporting the conclusions of this article will be made available by the authors, without undue reservation.

## Ethics Statement

The animal study was reviewed and approved by Institutional Animal Care and Use Committee, Office of Animal Welfare, University of Washington.

## Author Contributions

CC, WC, and JO designed the experiments and wrote the manuscript. CC and JO performed the experiments. CC, JO, and MB performed the analysis. All authors contributed to the article and approved the submitted version.

## Conflict of Interest

The authors declare that the research was conducted in the absence of any commercial or financial relationships that could be construed as a potential conflict of interest.

## Publisher’s Note

All claims expressed in this article are solely those of the authors and do not necessarily represent those of their affiliated organizations, or those of the publisher, the editors and the reviewers. Any product that may be evaluated in this article, or claim that may be made by its manufacturer, is not guaranteed or endorsed by the publisher.
